# Meningitis Caused by *Streptococcus agalactiae* in Nile Tilapia (*Oreochromis niloticus*): Infection and Inflammatory Response

**DOI:** 10.3390/ani10112166

**Published:** 2020-11-20

**Authors:** Silas Fernandes Eto, Dayanne Carla Fernandes, Alessandra Cristina de Moraes, João Victor da Costa Alecrim, Pedro Galdino de Souza, Fabíola Christian Almeida de Carvalho, Ives Charlie-Silva, Marco Antonio de Andrade Belo, João Martins Pizauro

**Affiliations:** 1 Department of Postgraduate in Health Sciences-PROCISA, Federal University of Roraima (UFRR), Boa Vista 69310-000, Brazil; joaovictoralecrim73@gmail.com (J.V.d.C.A.); pgpg919@gmail.com (P.G.d.S.); fabiola.carvalho@ufrr.br (F.C.A.d.C.); 2 Immunochemistry Laboratory, Butantan Institute, São Paulo 05503-900, Brazil; dayanne.fernandes@butantan.gov.br; 3 Department of Veterinary Medicine, Federal University of Rondônia (UNIR), Rondônia 76801-058, Brazil; alecris_moraes@hotmail.com; 4 Department of Pharmacology, Institute of Biomedical Sciences, University of São Paulo, ICB-USP, São Paulo 01222-010, Brazil; charliesilva4@hotmail.com; 5 Department of Preventive Veterinary Medicine of Unesp, School of Agrarian and Veterinary Sciences, Sao Paulo State University (Unesp), Jaboticabal 14884-900, Brazil; maabelo@hotmail.com; 6 Laboratory of Animal Pharmacology and Toxicology, University of Brazil, Descalvado 13690-000, Brazil; 7 Department of Technology, School of Agrarian and Veterinary Sciences, Sao Paulo State University (Unesp), Jaboticabal 14884-900, Brazil; j.pizauro@unesp.br

**Keywords:** streptococcosis, microglia, teleost fish, neuropathy

## Abstract

**Simple Summary:**

*Streptococcus agalactiae* (group B *Streptococcus*, GBS) is the main pathological agent in meningitis in Nile tilapia (*Oreochromis niloticus*). In this study, we describe the mechanism of infection and the immune response in the brain tissue of experimentally infected tilapia. This study understanding of the pathophysiology of meningitis in this species and bring the possibility of using tilapia as a study model for meningitis.

**Abstract:**

*Streptococcus agalactiae* (*Sta*) of Lancefield group B is the primary etiological agent of bacterial meningitis in Nile tilapia and newborn humans. Thus, the study of this disease is of fundamental importance for aquaculture and human medicine. Additionally, elucidation of the mechanisms involved in the host–pathogenic response is important for the success of new therapies. In the present study, we elucidated important aspects of the innate immune response in the brain tissue of Nile tilapia (*Oreochromis niloticus*) infected by *Sta*. The neuroinflammatory process in the meninges started with the migration of MHC class II and CD68 + cells, production of TNF-alpha, and the effective immune response to *Sta* was mediated by the increased iNOs+. In conclusion, the present study brings a partial understanding of the pathophysiological and neuroinflammatory mechanisms in meningitis in *Sta* infected tilapia, enabling important advances in the therapy of this disease as well as the possibility of using this biological model to understand human meningitis.

## 1. Introduction

*Streptococcus agalactiae* (*Sta*) is an important pathogen for animal and human health, with nine serotypes (Ia, Ib and II–XII) of this species having been identified to date [1]. Serotypes Ia, Ib and III infect Nile tilapia, while serotypes Ia, III, IV and V infect humans [2]. In addition to the similarities that serotypes of this species exhibit with respect to infection, a variant strain of serotype III-4 was isolated and identified from aquatic organisms that is associated with human infections in Asia, demonstrating the zoonotic potential and the imminent risk of this bacterium to the public health [3].

In addition, Nile tilapia (*Oreochromis niloticus*) is one of the most important commercial fish species worldwide [4], producing 4.5 million tonnes per year, generating an annual revenue of USD 7.5 billion [5]. In contrast, the economic losses due to *Sta* in world fish farming was 40 million dollars annually [6]. Representing a major threat to the fish farming industry, particularly the tilapia industry [7].

Meningitis is a characteristic clinical manifestation in Nile tilapia infected with *Sta*. Infected fish show severe neurological clinical signs, such as eradicated swimming, exophthalmos, corneal opacity, and histopathological examination shows suppurative meningitis. [8]. The ability of *Sta* to survive intracellularly in host macrophages and use these cells to transpose the blood–brain barrier and infect the meninges is the basis of the *Sta* infectious process. This process is dependent on virulence factors, including polysaccharide capsule (PC) and enzyme dismutase, which have physical and enzymatic mechanisms of action, respectively, in blocking phagolysosomal activity [9].

Microglia are phagocytic cells responsible for the innate immune defense in the brain, actively participating in the neuroinflammatory process through the production of proinflammatory cytokines, such as TNF-α, IL-1β, and IL-6 [10,11]. Although beneficial and necessary, the excessive production of these cytokines and, consequently, that of nitric oxide (NO) can result in severe tissue damage in the nervous tissue of the host [12].

Thus, the ability of *Sta* to infect multiple species and adapt to the different defense mechanisms among different aquatic or terrestrial species highlights the importance of validating representative experimental models used to study this pathogen. Such studies allow for an understanding of the virulence mechanisms of the pathogen and defense mechanisms of the host, which is fundamental for the development of new therapies for the neutralization of the pathogen and modulation of the neuro-inflammatory response.

## 2. Materials and Methods

### 2.1. Ethics Statement

All handling of fish was conducted in accordance with the National Council for Control of Animal Experimentation (CONCEA), and this study was approved by the ethics council (number: 17209/15).

### 2.2. Bacteria and Experimental Infection

*Sta* was isolated from tilapia and identified by the method described by El-Razika et al. [13]. The *Sta* strain (GenBank accession number: MH359095.1) was isolated from tilapia and cultured in brain heart infusion (BHI) broth (Difco, Detroit, MI, USA) at 28 °C with shaking for 48 h. The agar was removed by centrifugation at 10,000× *g* for 10 min at 4 °C, and the cell pellet was washed with 0.15 M PBS pH 7.2 four times, after which the cell density was readjusted to 2.0 × 10^5^ CFU/mL, with the lethal dose (LD50) having been predetermined following the recommendations of Eto et al., [14]. Forty Nile tilapia (84.3 ± 5 g) belonging to fish farming Pirajuba located in Porto Ferreira-SP/Brazil were randomly distributed in tanks with a capacity of 1500 L. In the experimental period, the water quality remained in the fish’s comfort range (OD: 7.5 ± 0.5 mg L^−1^; °C: 28.4 ± 0.9 °C; pH: 7.4 ± 0.3 and conductivity: 138.9 ± 12.6 S cm^−1^) A parameter probe (YSI Models 55–63, Chicago, IL, USA) was used. Fish were anesthetized by immersion in a benzocaine solution (Sigma-Aldrich Laboratory, Steinheim, Germany) (1:20,000 *v*/*v*) diluted in 98% ethanol (0.1 mg/mL), after which twenty fish (infected group) were challenged with the live *Sta* LD50, while the remaining twenty fish (control group) were given PBS alone. Fish were euthanized by immersion in benzocaine (1:500 *v*/*v*) period of seven days’ post infection, with brain tissue subsequently collected and fixed in 10% buffered formalin, processed in paraffin, sliced longitudinal cut to a thickness of 3 to 5 μm and mounted on slides for use.

### 2.3. Immunofluorescence and Immunohistochemistry

Direct immunofluorescence (IMF) was performed according to De Mateo et al. [15]. After tissue sections were deparaffinized and hydrated, antigen retrieval was performed under moist heat with citrate buffer (pH 6.0) for 10 min. Next, endogenous peroxidase and nonspecific sites were blocked for 1 h. The primary antibodies used were anti-MHC class II (dilution 1:500, Abmart/X1-H9B8H2), anti-CD68 (dilution 1:500, Abmart/X-I3KF76-N), anti-iNOS (dilution 1:500, Neomarkers/RB-1605-P) and anti-TNF-α (1:500 dilution, Anaspec/AS-55383) containing 1% bovine serum albumin (BSA, Sigma-Aldrich, St. Louis, MO, USA) were incubated by 2 h at room temperature, and the sample was washed twice in 0.05% PBS-Tween. The secondary rabbit anti-IgG antibody (Dako/K4061) diluted according to the manufacturer’s instructions. After incubating tissue samples with the appropriate antibodies, the IMF sample was washed and incubated with 4′,6-diamidino-2-phenylindole dihydrochloride (DAPI, Sigma Aldrich Corp, St. Louis, MO, USA), and the slide was mounted with Fluoromount for analysis and image capture using a fluorescence microscope (Olympus BX56 series, Olympus Life Science, Center Valley, PA, USA). The slides used for IHC with the substrate diaminobenzidine (DAB, Sigma-Aldrich, St. Louis, MO, USA) were assembled and recorded using the same microscope.

### 2.4. Image Analysis

Digital images of ten photomicrographs per antibodies (seven animals, *n* = 10) were captured using a digital camera (Olympus BX56 series, Olympus Life Science, Center Valley, PA, USA). The IHC images used were stained with DAB and hematoxylin. ImageJ software was used as described by Varghese et al., [16]. Positive staining for MHC class II, CD68+, TNF-α+ and iNOS+ cells was quantified using Color Deconvolution tools, and the results are presented as percentages of the area. This analysis allowed evaluation of the meningeal region of the brain. The IHC findings relative to the percentage of area were scored as low (2–4% positive cells/mm^2^), medium (6–8%) or high (10–12%).

### 2.5. Statistical Analysis

For the statistical analyses, immunostaining data are presented as mean values (*n* = 10). Comparisons of the three histological regions were performed using the ANOVA procedure, with the Statistical Analyses System (2001), and the normality of residuals was assessed for MHC class II, CD68, TNF-α and iNOS levels; each were analyzed separately to assure valid analyses. Significant differences (*p* < 0.05) were estimated using Tukey’s test, as described by Snedecor and Cochran [17].

## 3. Results

### 3.1. Infection

**Figure 1:**
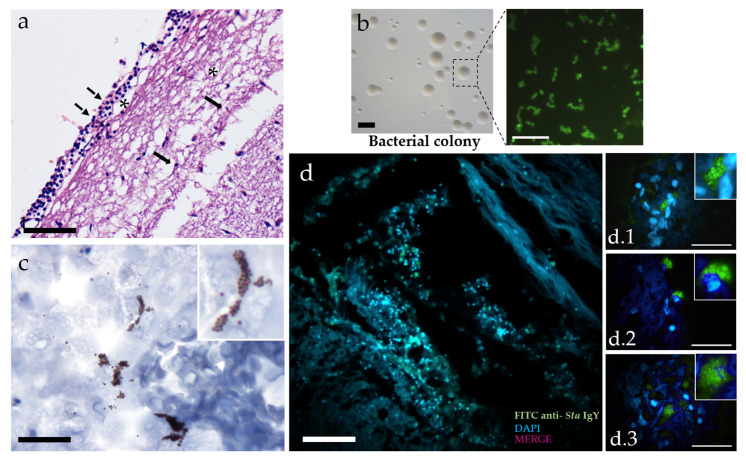
Mechanism of *S. agalactiae* (*Sta*) infection in the brain of Nile tilapia (*Oreochromis niloticus*). (**a**) Histopathological lesions in cerebral tissue of Nile tilapia infected with *Sta*. The dotted arrows indicate leukocyte infiltrates, asterisks indicate suppurative lesions, and arrows indicate microglial migration. (**b**) Microbiological identification by immunofluorescence of *Sta* isolated from the brain of tilapias 7 days after infection. (**c**) Detection of *Sta* in brain tissue by IHC (**c**) immunofluorescence (IMF) (**d**) *Sta* in the light of meningeal endothelium (**d.1**) intracellular phase without phagocyte (**d.2**) and, free form colonies of *Sta* in the interstice of brain tissue (**d.3**). Bar = 25–50–100 μm: hematoxylin and eosin staining (H&E); IMF stain: DAPI = nucleus (blue) and fluorescein isothiocyanate (FITC) = IgY anti-*S. agalactiae* (green).

Seven days after infection with LD50, the fish presented clinical signs of neurological diseases, such as eradication and exophthalmos, characterizing an infection by *S. agalactiae*. The histopathological analysis shows the leukocyte infiltrate in the meninges (Figure 1a). The etiologic agent identification by direct immunofluorescence of the colonies shown in (Figure 1b). IHC confirmed *Sta* colonies in the brain (Figure 1c).

In Figure 1d, we observed the infection, showing the entry via *Sta* meningeal endothelium (Figure 1d.1) in the intracellular phase without phagocyte (Figure 1d.2) and, free form colonies of *Sta* in the interstice of brain tissue (Figure 1d.3).

### 3.2. Neuroinflammation

**Figure 2:**
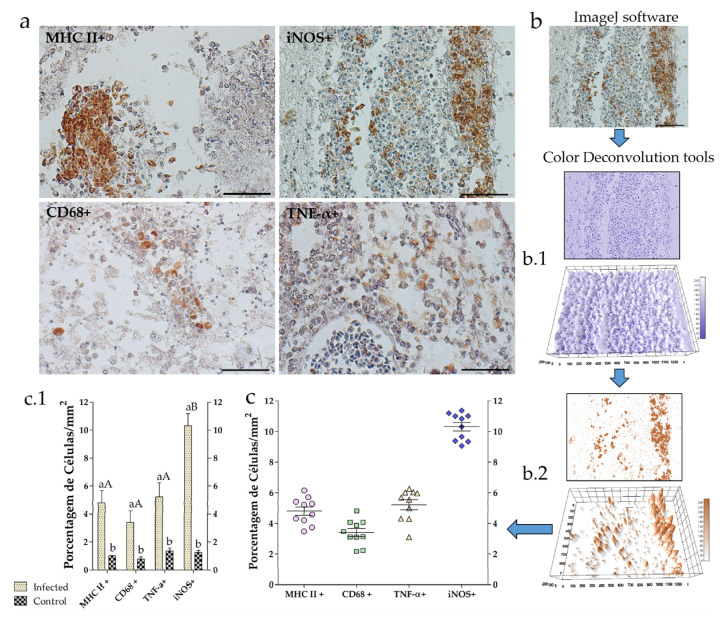
Neuroinflammatory process in the brain of Nile tilapia (*Oreochromis niloticus*) infected with *S. agalactiae*. (**a**) Representative images of the distribution of MHC class II+, CD68+, TNF-α and iNOS+ cells in meningeal. (**b**) Software ImageJ, using Deconvolution tools that separate the image in nuclear bodies (hematoxylin) (**b.1**) and the immunostaining in DAB (brown) (**b.2**) and (**c**,**c.1**) the quantification analysis. Means and respective standard deviations (*n* = 10) are shown; different letters indicate significant differences (Tukey’s test, *p* < 0.05) between the observed histologically stained regions; ns = not significant. Bar = 50 μm, DAB = immunostaining (brown), counter stained with Harris hematoxylin.

The neuroinflammatory process was initiate by MHC II + and CD68 + cells migration, production of TNF-α and iNOS in meninges (Figure 2a). Figure 2b shows the flowchart of the morphometric technique micrograph analyzed by Software ImageJ, using deconvolution tools that separate the image in nuclear bodies (hematoxylin) (Figure 2b.1) and the immunostaining in 3′-diaminobenzidine (DAB) in brown (Figure 2b.2) calculating the percentage of the immunostained area (Figure 2c). Subsequently, the percentage of MHC II + CD68 + cells, differing only from the control group as well as TNF-α and only the production of iNOS had a significant increase (*p* < 0.05) in the meninges. The control group showed baseline levels for all markers, with a non-significant percentage (Figure 2c,c.1).

## 4. Discussion

Meningitis caused by *Sta* in Nile tilapia and its importance for aquaculture health has been previously described [18,19,20]. In humans *Sta* is the main microorganism that causes bacterial newborn meningitis [21]. Responsible for infecting 0.2 to four cases per 1000 newborns worldwide [22]. Since *Sta* has become a commensal and opportunistic pathogen [23], new therapeutic strategies are needed to control infections. However, to assess the effectiveness of these new methods, it is important to understand the mechanisms involved during host–pathogen interactions in representative biological models. Although mammalian models such as rats and mice are widely used to study the pathophysiology of *Sta* [24,25]. In recent years, aquatic organisms have gained prominence as a study model for infectious diseases in particular zebrafish using in neurodegenerative studies [26] and in *Sta* infection [27].

In previous studies, our laboratory has substantiated the use of Nile tilapia as a study model for meningitis caused by *Sta* [14] *Aeromonas hydrophila* [28] and for acute inflammatory study [29]. Nurani et al., [30] proved the effectiveness of using a polyvalent vaccine for *Sta* and the ability to passively transfer to offspring. In addition to the importance of this study for aquaculture health, these findings may collaborate as a model for the prophylaxis of human neonatal meningitis. However, the mechanisms involved in the pathophysiology, inflammation and innate immune defense of the brain for this disease are not well elucidated for this species, and complementary information is needed.

In this study, the experimental infection of Nile tilapia by *Sta* and the viability of the bacterium in the host tissue were confirmed by observations of clinical neurological signs, microbiological identification assays and histopathological lesions. The IMF results show that *Sta* cells were internalized by macrophages near the meningeal vascular endothelium, allowing bacteria to enter the meninges and for free-living bacterial cells to colonize the meninges. This result in particular supports the hypothesis of the intracellular survival of *Sta* in macrophages and their use of these cells to transpose the blood–brain barrier and infect the nervous system of the host. This mechanism is called a “Trojan horse” and is used by other *Streptococcus* strains belonging to Lancefield group B to infect mammals [31] and humans [32]. Although intracellular survival of *Sta* in fish macrophages has been described in vitro [33], the ability of these infected cells to migrate to the central nervous system has not been fully elucidated.

Immunohistochemistry differs from other immunological techniques in that it allows a specific epitope to be detected, revealing its location in the tissue and allowing for functional studies of the host response [28]. Thus, the neuroinflammatory process in the brain of tilapia infected with *Sta* began with the infiltration of MHC class II and CD68 positive cells in meningeal. The expression of MHC class II proves the ability of these cells to present antigens and characterize them as functional leukocytes [34]. CD68 is a lysosomal membrane marker that features activated phagocytes and in a phagocytic process [35]. The migratory pattern of these cells seen in the tilapia brain tissue was similar in the homologous tissue of humans who died of sepsis [36]. Jiang-Shieh et al. [37] showed that the lipoteichoic acid (LTA) present in the cell wall of Gram-negative bacteria increased the expression of MHC class II, complement receptor type 3, CD14 molecule, and macrophage scavenger receptor. In addition, the same author reports that LTA increased the expression of inducible nitric oxide synthase in microglia, which will be discussed later.

TNF-α plays a role in cell differentiation, NO production, and stimulation of the expression of other pro-inflammatory cytokines and has vasodilatory and autocrine effects in microglia differentiation [38,39]. In this study, infection of the tilapia meninges by *Sta* induced the production of TNF-α by phagocytes, initiating the neuroinflammatory process in the brain tissue. This fact is justified since neurons and glial cells also produce TNF-α [39]. In addition, the cell location of the tumor necrosis factor (TNF-α) converting enzyme (TACE) in the human brain was analyzed by immunohistochemistry and its expression was detected in different neuronal populations, including pyramidal neurons of the cerebral cortex and neurons of the layer granular cell in the hippocampus [40,41]. Thus, we can say that the kinetics of TNF-α production in our study was similar to human and mammalian models.

Allan et al. [42] suggest that infection by group B *Streptococcus* in the lung tissue induces the production of nitric oxide and pro-inflammatory mediators. Leib et al. [43] explored the role of synthetic inducible nitric oxide (iNOS) in an infant rat model for group B streptococcal meningitis and observed the increased iNOS during meningitis mainly on the walls of the meningeal vessels. Our results were similar, and the production of iNOS seems to be strictly controlled, being produced in excess only in locations associated with pathogens, participating in a phagocytic activity and vascular changes in the endothelium of the meningeal vessels. The same authors mentioned above showed that the use of an iNOS inhibitor aminoguanidine in meningitis increased the bacterial titers in the cerebrospinal fluid and the incidence of seizures compared to untreated infected animals [44]. However, although physiologically necessary and beneficial to the host, excessive production of NO can lead to irreversible tissue damage in the host brain tissue [45]. Thus, control over the production of this reactive agent observed in the cerebral tissue of tilapia during infection by *Sta* is beneficial and necessary.

## 5. Conclusions

In conclusion, we demonstrate that the innate immune response mechanisms during the neuroinflammatory reaction caused by *Sta* infection in Nile tilapia are similar to those described during meningitis caused by other *Streptococcus* of Lancefield group B in mammals, making this species a representative biological model for the study of this disease. Our study illustrates the mechanisms of infection, neuroinflammation and immune response in the brain tissue of Nile tilapia (*Oreochromis niloticus*) infected with *S. agalactiae*.

## Data Availability

Data are available upon request to the corresponding author.
